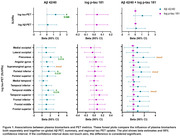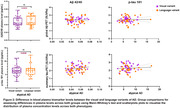# Aβ42/40 and p‐tau 181 as disease biomarkers in Atypical Alzheimer’s disease

**DOI:** 10.1002/alz.091571

**Published:** 2025-01-09

**Authors:** Neha Atulkumar Singh, Jonathan Graff‐Radford, Danni Li, Michelle M. Mielke, Mary M. Machulda, Christopher G. Schwarz, Matthew L. Senjem, Clifford R. Jack, Val J. Lowe, Keith A. Josephs, Jennifer L. Whitwell

**Affiliations:** ^1^ Mayo Clinic, Rochester, MN USA; ^2^ University of Minnesota, Minneapolis, MN USA; ^3^ Wake Forest University School of Medicine, Winston‐Salem, NC USA

## Abstract

**Background:**

Blood plasma metrics provide excellent biomarkers of the presence of Alzheimer’s disease (AD) pathology in population cohorts. However, it is less clear whether plasma metrics correlate with PET measures of AD pathology in cohorts of patients with advanced AD disease, particularly in those with atypical clinical presentations of AD.

**Method:**

Seventy‐seven patients (visual variant=43, language variant=32, motor variant=1 and dysexecutive variant=1) with PET biomarker‐confirmed atypical AD were recruited by the Neurodegenerative Research group at Mayo Clinic, Rochester, MN, underwent Aβ (Pittsburgh Compound‐B) and tau (^18^F‐flortaucipir) PET and provided a blood sample. Blood plasma analysis was performed to assess p‐tau 181 and Aβ42/40 ratio concentrations using the Quanterix Simoa platform. Multivariate linear regression models assessed the relationship between plasma concentrations and both global Aβ, atypical AD summary and regional tau PET measures. For each PET measure, two separate models were fit to assess the influence of each plasma biomarker separately, while the third model included all plasma measures to assess the combined influence of all biomarkers on PET uptake. To investigate differences in plasma concentrations between atypical AD phenotypes, we performed Mann‐Whitney’s test across the visual and language variants of AD.

**Result:**

Plasma Aβ42/40 ratio showed positive associations with tau PET uptake, specifically within precuneus, parietal and temporal regions and was not associated with Aβ PET. There was no relationship between plasma p‐tau 181 and Aβ or tau PET uptake. However, the model that included both plasma biomarkers showed that with the addition of p‐tau 181, all significant associations between Aβ42/40 ratio and regional tau uptake were weakened to trends. The Aβ42/40 ratio continued to show no relationship with global Aβ‐PET uptake and plasma p‐tau 181 showed no relationship to PET measures (Figure 1). Lastly, no significant difference in plasma Aβ42/40 and p‐tau 181 levels was noted between the visual and language variants of AD (Figure 2).

**Conclusion:**

The lack of associations between plasma concentrations and PET measures of Aβ and tau in this relatively advanced AD cohort may suggest that plasma concentrations have greater utility as diagnostic biomarkers of early AD as compared to advanced AD.